# Mitigating the Agglomeration of Nanofiller in a Mixed Matrix Membrane by Incorporating an Interface Agent

**DOI:** 10.3390/membranes11050328

**Published:** 2021-04-29

**Authors:** Manh-Tuan Vu, Gloria M. Monsalve-Bravo, Rijia Lin, Mengran Li, Suresh K. Bhatia, Simon Smart

**Affiliations:** 1 School of Chemical Engineering, The University of Queensland, Brisbane, QLD 4072, Australia; manh.vu@uq.net.au (M.-T.V.); g.monsalvebravo@uq.edu.au (G.M.M.-B.); m.li6@uq.edu.au (M.L.); s.bhatia@uq.edu.au (S.K.B.); 2 Institute for Tropical Technology, Vietnam Academy of Science and Technology, Hanoi 100000, Vietnam; 3 Dow Centre for Sustainable Engineering Innovation, The University of Queensland, Brisbane, QLD 4072, Australia

**Keywords:** nanodiamond, mixed matrix membrane, filler dispersion, Felske model, gas separation

## Abstract

Nanodiamonds (ND) have recently emerged as excellent candidates for various applications including membrane technology due to their nanoscale size, non-toxic nature, excellent mechanical and thermal properties, high surface areas and tuneable surface structures with functional groups. However, their non-porous structure and strong tendency to aggregate are hindering their potential in gas separation membrane applications. To overcome those issues, this study proposes an efficient approach by decorating the ND surface with polyethyleneimine (PEI) before embedding it into the polymer matrix to fabricate MMMs for CO_2_/N_2_ separation. Acting as both interfacial binder and gas carrier agent, the PEI layer enhances the polymer/filler interfacial interaction, minimising the agglomeration of ND in the polymer matrix, which is evidenced by the focus ion beam scanning electron microscopy (FIB-SEM). The incorporation of PEI into the membrane matrix effectively improves the CO_2_/N_2_ selectivity compared to the pristine polymer membranes. The improvement in CO_2_/N_2_ selectivity is also modelled by calculating the interfacial permeabilities with the Felske model using the gas permeabilities in the MMM. This study proposes a simple and effective modification method to address both the interface and gas selectivity in the application of nanoscale and non-porous fillers in gas separation membranes.

## 1. Introduction

Membranes are recognised as an energy-efficient and environmental-friendly separation technology for gas separation. However, most of the conventional polymer membranes are restricted to a trade-off between mass transport rates and separation efficiency, while inorganic membranes face the limit of poor scalability and high cost [1,2]. Mixed matrix membranes (MMMs), which can be prepared by embedding selective filler materials into a continuous polymer matrix, offer an opportunity to overcome the drawbacks associated with pure polymer or inorganic membranes [3,4,5,6]. With proper selection of the filler/polymer pair, mixed matrix membranes benefit from both the ease of processing of the polymer materials and superior separation properties of the filler particles. The fabrication of MMMs is analogous to that of pure polymer membranes, which means that MMMs could be manufactured on industrial scales by modifying existing polymer membrane production technologies [7].

Pair-wise selection of the filler and polymer is the basis for the MMM fabrication because it is relevant to the morphology and performance of the membrane. The selection of polymer determines the minimum gas transport performance (diffusivity and solubility) of the membrane. The embedded filler defines the improvement of the gas permeability and selectivity of the MMM [7]. Generally, nano sized fillers are preferred due to their relatively good dispersion in the polymer matrix and the strong interaction with the polymer phase.

Nanodiamonds (NDs) are specific carbon nano-crystalline particles with a tetrahedral diamond structure [8]. ND particles have primarily a spherical shape with an average diameter of 5–10 nm with a narrow particle size distribution. NDs are potential candidates for various applications, such as composite materials [9,10], quantum sensing and imaging [11,12], and biomedical applications [13,14], owing to NDs’ chemical stability, thermal conductivity, biocompatibility, hardness, and photoluminescence properties [15,16,17].

Recently, NDs have been applied as the nanofiller into several polymer matrices for the fabrication of polymer composites. The incorporation of ND can improve the strength, thermal stability and conductivity, electrical conductivity, chemical resistance and biocompatibility of the polymer matrices [18,19]. Functionalised ND was embedded into epoxy by in situ polymerization. The incorporation of the ND resulted in high thermal stability and thermal conductivity of the epoxy composite compared to the pure polymer matrix [20]. Etemadi et al. introduced amino-functionalised ND into cellulose acetate membrane for anti-biofouling in membrane bioreactor. The incorporation of amino-functionalised ND improves the hydrophilicity of the membrane [21]. Carboxylated ND was prepared by Li et al. and embedded in polyvinylidene fluoride. The polyvinylidene fluoride membrane blended with carboxylated ND showed higher hydrophilicity and water permeability, as well as improved anti-fouling performance [22]. A composite membrane based on polyvinylidene fluoride/nanodiamond has been synthesised for better desalination performance [23]. Nanodiamond was firstly introduced in an interfacial polymerisation system for the fabrication of nanofiltration membranes [24]. The introduction of ND transforms the membrane structure from nodules to ridges at the nanoscale. The resulted nanofiltration membrane presented a high-water permeability and salt rejection. Other work by Polotskaya et al. selected ND as an inorganic filler for poly(2,6-dimethyl-1,4-phenylene oxide) membranes for gas separation [25]. The same group also incorporated ND into polyphenylene-isophthalamide matrix to prepare MMMs for gas separation [26]. Their results showed that the selectivity of H_2_/N_2_ and O_2_/N_2_ gas pairs increased with the ND concentration up to 3 wt.%.

The dispersion of filler particles in the continuous polymer phase is vital to the performance of the MMMs. However, NDs tend to form aggregates spontaneously to minimise surface energy [24]. The particle agglomeration can lead to the formation of non-selective interfacial voids inside the particle aggregates, which can cause the decline of membrane gas selectivity. The most common approach to improve filler dispersion is the priming method. In this approach, the filler particles are coated with a thin layer of the polymer by introducing a small amount of polymer into the filler suspension, before mixing it with the remaining bulk of the polymer. The priming method can decrease stress at the filler/polymer interface, minimising the aggregation of the fillers [27]. Nevertheless, applying the priming process by itself will not eliminate the formation of particle agglomeration in MMMs with nanoparticles. The dispersion of inorganic fillers can also be improved by applying the interfacial polymerisation processes [28]. In this approach, the inorganic filler is dispersed along with the organic monomer and the polymerisation will occur on the interface between filler and monomer.

In this study, polyether block amide Pebax (MH 1657) was chosen as the polymer matrix for the MMM fabrication due to its excellent CO_2_ selectivity [29]. Pebax consists of polyamide (PA) as hard segments and polyether (PE) as soft segments in the polymer chains, where the hard crystalline PA block provides mechanical strength and the soft polyether block plays as the gas permeable phase due to its high chain mobility [30]. Nanodiamond was chosen as the filler for MMM fabrication. We propose here an effective and convenient route for mitigating the agglomeration of NDs in MMM by incorporating a third interface agent. Controllable decoration of low molecular weight polyethyleneimine (PEI) on NDs was performed before embedding into the polymer matrix. PEI acts as a wetting and binding material between ND and polymer, as well as a CO_2_ carrier agent. Focused ion beam scanning electron microscopy (FIB-SEM) was applied to provide a quantitative analysis of the distribution of ND aggregates within the polymer matrix. Improved dispersion of NDs has been achieved with the incorporation of PEI and the size and quantity of the ND aggregates are significantly reduced. The CO_2_/N_2_ selectivity of Pebax/ND-PEI MMMs have been improved compared to the Pebax/ND MMMs and pristine Pebax membranes. Finally, such an enhancement of the polymer/filler interfacial properties is corroborated by calculating the interface thickness and gas permeability with the Felske model [31,32,33,34] using the CO_2_/N_2_ Pebax/ND-PEI MMM permeabilities.

## 2. Materials and Methods

### 2.1. Materials

Nanodiamond (≥97% trace metals basis, particle size < 10 nm) and polyethyleneimine (PEI, average M_n_~1200, average M_w_~1300) were purchased from Sigma-Aldrich. The received nanodiamond powder was heated at 425 °C in the air in a furnace for 4 h (heating rate 10 °C/min) to maximise the oxygen functional groups on the surface of ND [35]. Pebax (MH 1657) was kindly supplied by Arkema, France. Ethanol was obtained from Merck. All the chemicals were used without further purification. Cylinders of pure CO_2_ and N_2_ were supplied by Coregas.

### 2.2. Nanodiamond Surface Modification

An amount of 2 g of polyethyleneimine (PEI) was dissolved in 15 mL of deionised water and stirred in 30 min to form a homogeneous solution. 0.2 g of ND was then dispersed into the PEI contained solution and sonicated for 15 min in order to completely dispersed the ND nanoparticles. The well-dispersed mixture was subsequently heated to 70 °C whilst stirring at 250 rpm for 24 h. The mixture was then centrifuged and washed with deionised water for at least three times before drying in a vacuum oven at 100 °C for 24 h and stored under vacuum before use. The obtained sample was then labelled as ND-PEI.

### 2.3. Fabrication of Nanodiamond Incorporated Mixed Matrix Membranes

For the neat Pebax membrane, 0.48 g of Pebax was dissolved in a mixture of ethanol/water (70 wt.%/30 wt.%) by heating up to 70 °C and stirring for 6 h. The resulting solution was then cast on a flat glass surface at 30 µm of thickness and dried in a vacuum oven at 100 °C in 24 h. The obtained membrane was then peeled off the glass plate and stored under vacuum before use.

For the mixed matrix membrane fabrication, a quantity of nanodiamond (ND or ND-PEI) was dispersed into a mixed solution of ethanol/water (70 wt.%/30 wt.%) and sonicated for 15 min. Pebax was then slowly added into the mixture while heating to 70 °C along with sonication several times during the process. The amount of Pebax and nanodiamond was calculated in order to form the ratio: Pebax/ND = 99.9/0.1, 99.5/0.5, 99/1, 98.5/1.5, referred to as the nominal ND loading in Section 3. The resulting mixture was cast onto a clean glass plate at a thickness of 30 µm and dried at 100 °C in 24 h before peeling off. The thickness of pure Pebax and MMMs were measured using a micrometre within the range of 40−50 μm. The membranes were stored with desiccant under vacuum before gas permeation tests and characterisation.

### 2.4. Characterisation

The content of carbon, hydrogen and nitrogen in ND and ND-PEI samples was quantified by a Thermo Scientific^TM^ FLASH 2000 CHNS/O Analyzer. X-ray photoelectron spectrometer (XPS) was applied by a Kratos Axis Ultra XPS equipped with a 165 mm hemispherical electron energy analyser and a monochromatic Al Kα (1486.6 eV) radiation at 150 W (15 kV, 10 mA). The C1s main peak position was fixed at 284.8 eV and taken as an internal standard. The loading of PEI on the ND particles was calculated by the Perkin Elmer Instruments STA 6000 Thermo Gravimetric Analyser. The ND-PEI sample was heated under an air atmosphere at a uniform heating rate of 5 °C min^−1^ from 40 to 800 °C. Fourier-transform infrared (FTIR) spectra of ND, ND-PEI and PEI were obtained by using a Perkin Elmer Spectrum 100 spectrometer equipped with an attenuated total reflection (ATR) objective. 

Transmission electron microscopy (TEM) was performed on a Tecnai 20 FEG TEM with the accelerating voltage of 200 kV. A JEOL JSM7100 scanning electron microscope (SEM) was applied for the morphologies of the MMM cross-sections. Focused ion beam scanning electron microscopy (FIB-SEM) was applied to evaluate the dispersion of nanodiamond in the Pebax matrix by using an FEI SCIOS FIB/SEM dual beam system. The description of the method is shown in our previous studies [36,37,38,39]. A hole was milled on the surface of the MMM by Ga^+^ FIB (Figure S1). Slices with a thickness of 10 nm were removed from the sample up to a depth of 10 μm by the Ga^+^ FIB at 30 kV and 3 nA. Back-scattered electron (BSE) imaging mode was applied to capture the exposed cross-section images automatically. A set of cross-section SEM images was captured during this slice-and-view section with resolution of 10 nm. The stack of these SEM images was aligned. The segmentation of different phases was identified by image thresholding. The ND fillers phase is the brightest as there is a trace of heavier elements (Na, Si, S) in NDs than Pebax. The dark phase corresponds to the polymer matrix. The stack of obtained SEM images was reconstructed in three-dimensions by using the software Avizo. The volume fraction of each phase was also calculated by the software.

### 2.5. Gas Permeation Test

The gas permeation of membranes was measured in a system with variable feed pressure and constant volume, as reported elsewhere [40]. The membranes were degassed to desorb the prior permeate gas before each test fully. The tests were carried out with 2 bar feed pressure and 0.015 in the downstream at 35 °C. The diameter of the effective permeation area was 0.935 cm. The permeability is calculated by the equation below:

(1) $$P=\frac{273.15\times 10^{10}}{760AT}\frac{VL}{\frac{P_{0}\times 76}{14.7}}\frac{dp}{dt}$$

where *P* is the permeability in barrer (1 barrer = 1 × 10^−10^ cm^3^ (STP) cm cm^−2^ s^−1^ cm Hg^−1^), A is the permeation area (cm^2^), T is the operation temperature (K), *V* is the dead volume of the permeate side (cm^3^), *L* is the thickness of the membrane (cm), *P*_0_ is the feed pressure (psi), and d*p*/d*t* is the steady rate of pressure increase in the permeate side (mm Hg s^−1^).

The ideal selectivity for two gases is calculated by:

(2) $$\alpha =\frac{P_{A}}{P_{B}}$$

where *P*_A_ and *P*_B_ are the permeability of pure gas A and B, respectively. Values and error bars reported in the figures and tables are based on measurements of three different membranes. 

### 2.6. Felske Model

In this work, the Felske model is used to estimate the filler-polymer interfacial properties (e.g., interface thickness and permeability) based on the the CO_2_/N_2_ experimental permeabilities. While detailed derivation of this model is presented in Ref. [31]. The model equations are described in the context of this work in what follows. Thus, the MMM permeability $\left(P_{m}\right)$ is defined as:

(3) $$P_{m}=P_{c}\left[\frac{2(1-\phi_{gc})+(1+2\phi_{gc})(\eta /\gamma )}{\left(2+\phi_{gc})+(1-\phi_{gc})(\eta /\gamma \right)}\right]$$

in which $\phi_{gc}=\phi_{f}+\phi_{i}$ is the volume fraction of total dispersed phase (i.e., filler particle and interfacial layer) following [41].

(4) $$\phi_{gc}=\frac{\phi_{f}^{N}}{\phi_{f}^{N}+\frac{\left(1-\phi_{f}^{N}\right)}{{\left(1+l_{i}/r_{o}\right)}^{3}}}$$

with $\phi_{f}^{N}=\phi_{f}/\left(\phi_{f}+\phi_{c}\right)$ being the nominal filler volume fraction, $l_{i}$ the interface thickness and $r_{o}$ the particle radius. The nominal filler volume fraction $\left(\phi_{f}^{N}\right)$ in the MMM is calculated from the nominal filler weight fraction $\left(\omega_{f}\right)$ as [26]:

(5) $$\phi_{f}^{N}=\frac{\omega_{f}}{\omega_{f}+\frac{\rho_{f}}{\rho_{c}}(1-\omega_{f})}$$

where $\rho_{f}=3.5\;g/\mathrm{mL}$ and $\rho_{c}=1.14\;g/\mathrm{mL}$ are the nanodiamond (particle) and Pebax densities, respectively. Further, η and γ in Equation (3) are given by [31].

(6) $$\eta =\left[2+{\left(1+l_{i}/r_{o}\right)}^{3}\right]\alpha_{fc}-2\left[1-{\left(1+l_{i}/r_{o}\right)}^{3}\right]\alpha_{ic}$$

(7) $$\gamma =\left[1+2{\left(1+l_{i}/r_{o}\right)}^{3}\right]-\left[1-{\left(1+l_{i}/r_{o}\right)}^{3}\right]\alpha_{fi}$$

where $\alpha_{fc}=P_{f}/P_{c}$, $\alpha_{ic}=P_{i}/P_{c}$ and $\alpha_{fi}=P_{f}/P_{i}$. In Equations (3)–(7), P and ϕ denote the permeability and volume fraction, respectively, and superscripts/subscripts f, c, i, m and g denote filler phase, continuous phase, interface, MMM, and combined filler phase and interface composite, respectively.

## 3. Results and Discussion

### 3.1. Preparation and Characterisation of Modified Nanodiamonds

**Figure 1:**
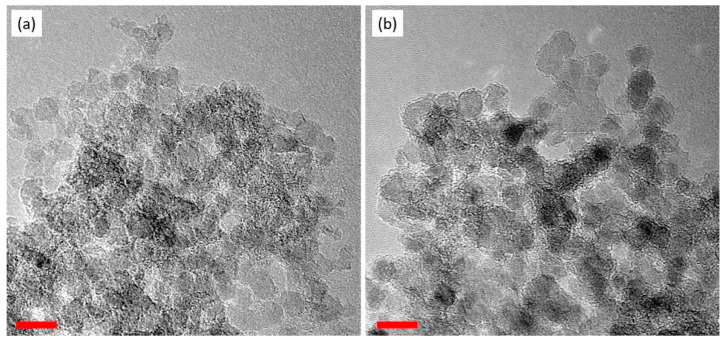
TEM images of (**a**) ND and (**b**) ND-PEI. The red lines are the scale bars. Both scale bars are 10 nm.

Figure 1 shows the TEM images of ND and ND-PEI. The ND expresses a spherical shape with a size of 5–10 nm. After the PEI impregnation, the size and shape of the ND particles did not change significantly. The loading of PEI in ND-PEI was calculated by TGA. Two main degradation stages were observed in the TGA result (Figure S2). The first stage of degradation from 150 °C to 350 °C should correspond to the degradation of PEI on the ND. The loading of PEI in ND-PEI is around 10%. The content of carbon, hydrogen and nitrogen in ND and ND-PEI samples has been conducted by an elemental analyser to confirm the success of the surface modification of ND by PEI and the results are shown in Table 1. Compared to the ND, the ND-PEI particles show a higher ratio of nitrogen and hydrogen elements, as would be expected under successful coating. The introduction of PEI layer onto the ND surface increased the amount of N and H elements. These results suggest that the PEI has been successfully incorporated on the ND surface.

**Figure 2:**
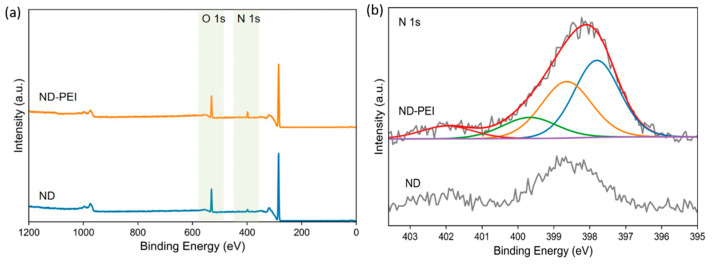
XPS spectra of ND and ND-PEI. (**a**) Survey scans the ranging from 0 to 1200 eV. (**b**) N1s.

X-ray photoelectron spectra (XPS) also has been applied to confirm the PEI layer coated on the ND. Figure 2a shows the survey scans of the ND and ND-PEI samples. ND-PEI shows higher (4.86 at%) nitrogen ratio than ND (1.83 at%), which is consistent with the elemental analysis results. For the N1s spectra (Figure 2b), the ND sample exhibited two peaks at around 397.8 and 402.0 eV, which correspond to the heteroatom nitrogen on the ND surface structure [42,43]. For the ND-PEI sample, four peaks were found at 397.8, 398.6, 399.7, and 402.0 eV. The two peaks found in ND spectrum at 397.8 and 402.0 eV also appeared in the spectrum of ND-PEI. The two new peaks at 398.6 and 399.7 eV correspond to amine and protonated amine groups in PEI chains, respectively. These XPS results demonstrate NDs are covered by the PEI. Fourier-transform infrared (FTIR) spectra of ND, NE-PEI and PEI were investigated (Figure S3). Compared with the spectrum of ND, the decrease of the peak at 1629 cm^−1^ (O-H bending, hydroxyl) and the increase of the peak at 1662 cm^−1^ (C=O stretching of amide) [44] were observed in the spectrum of ND-PEI, revealing the presence of PEI and its interaction with the oxidised surface of ND through the surface –OH groups.

### 3.2. Characterisation of MMMs

**Figure 3:**
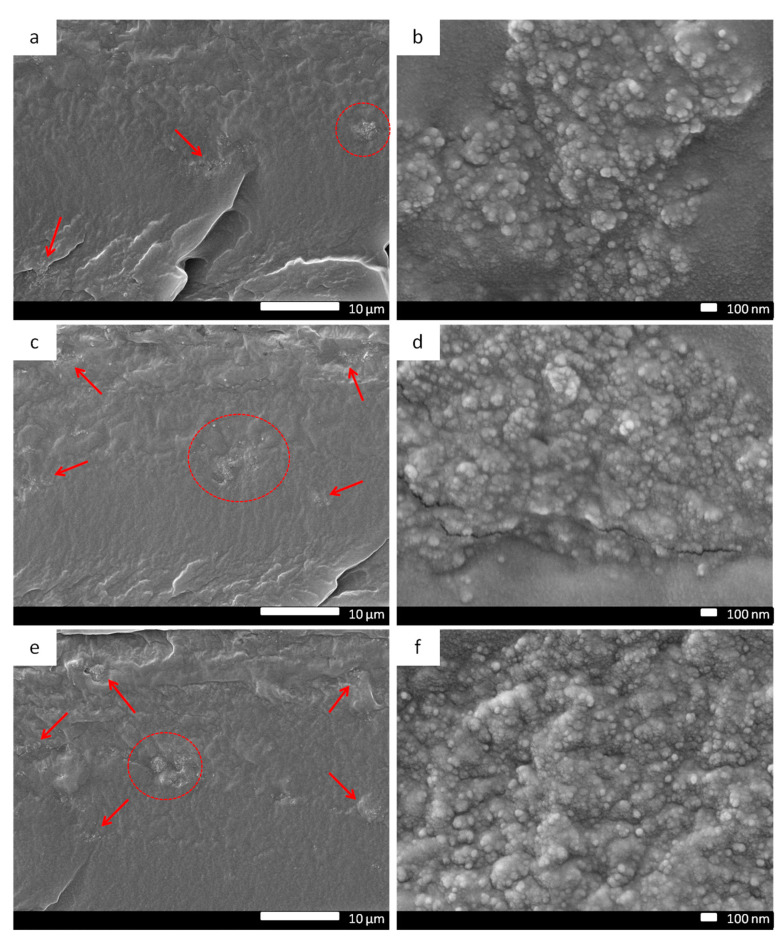
Membrane cross-section SEM images of Pebax/ND MMMs with different ND ratio: 0.1 wt.% (**a**,**b**), 0.5 wt.% (**c**,**d**), 1.0 wt.% (**e**,**f**), arrows point to ND aggregate clusters in the MMMs.

**Figure 4:**
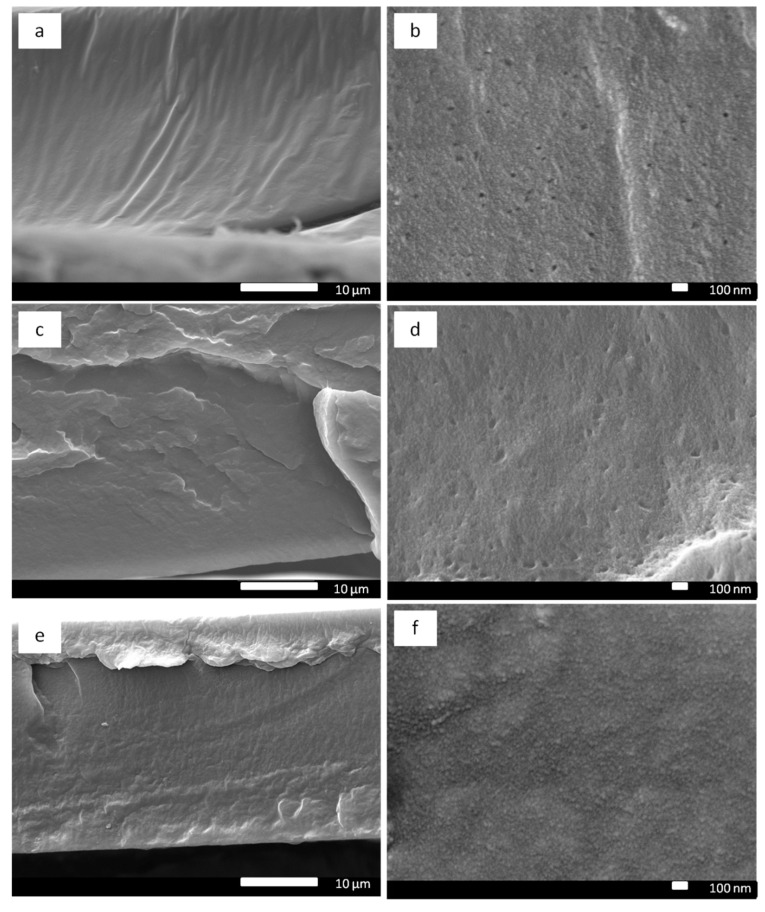
Membrane cross-section SEM images of Pebax/ND-PEI MMMs with different ND ratio: 0.1 wt.% (**a**,**b**), 0.5 wt.% (**c**,**d**), 1.0 wt.% (**e**,**f**).

The transport performance and the integrity of the membrane rely on the filler dispersion and the filler/matrix adhesion. The interfacial morphology of Pebax/ND MMMs and Pebax/ND-PEI MMMs were investigated by FESEM and the images are displayed in Figure 3 and Figure 4. Compared to the pristine Pebax membrane (Figure S4a,b), poor dispersion of ND in the Pebax matrix was clearly observed in the Pebax/ND MMMs (Figure 3), as indicated by large aggregates of ND (identified by arrows) in the matrix. It seems that the oxygen-containing functional groups on the ND surface do not provide appreciable assistance in dispersing the ND in the Pebax matrix. Without further surface modification, the ND tends to form agglomerations easily. The aggregation of ND can cause the formation of non-selective interfacial voids, leading to the deterioration in gas selectivity of the membrane. With further increases in the ND loading, unsurprisingly, more aggregate clusters with larger sizes were observed (Figure S4c,d).

In the case of Pebax/ND-PEI MMMs (Figure 4 and Figure S4e,f), the individual ND-PEI particles were less visible in the matrix. The roughness of the Pebax/ND-PEI MMMs was significantly increased compared to the neat Pebax membranes. In an obvious contrast to the Pebax/ND MMMs (Figure 3), there was no visible ND agglomeration at the cross-section of the Pebax/ND-PEI MMMs even with the highest loading of ND-PEI (1.5 wt.%) (Figure S4e,f). These SEM images indicate an improved dispersion of ND in polymer matrix was achieved. The improvement in dispersion of ND-PEI with the polymer matrix is attributed to the presence of the PEI layer on ND surface. The PEI layer acts as a polymeric compatibiliser, which can improve the compatibility between the crystalline carbon structure of ND and the rubbery polymeric nature of Pebax, preventing the filler aggregation and leading to an increase in dispersibility of ND in the Pebax matrix.

**Figure 5:**
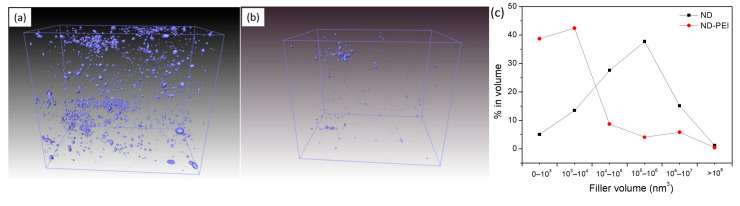
The FIB surface rendered view of (**a**) Pebax/ND MMM, (**b**) Pebax/ND–PEI MMM, and (**c**) filler particle size distributions as derived from image analysis of the FIB-SEM tomogram.

To further achieve more comprehensive structural information of the ND MMMs, the distribution of ND and ND-PEI particles within the Pebax matrix was investigated by focused ion beam SEM (FIB-SEM). Figure 5a,b shows the 3D reconstructed images of the filler distribution in Pebax/ND and Pebax/ND-PEI MMMs. As also observed in the SEM images above (Figure 3), the ND particles formed large agglomerates in the MMMs, while in contrast, ND-PEI aggregates were far less likely to be observed. Due to the presence of PEI, ND-PEI exhibit better dispersion than unmodified ND, presented as much smaller particles and aggregates. Besides, as the resolution of FIB observation is 10 nm, ND and ND-PEI particles with the size of 10 nm or below are hardly detected, which leads to fewer ND particles observed in the FIB images. Based on the 3D image analysis, the ND agglomerates size distributions in the MMMs were calculated and reported in Figure 5c. For the Pebax/ND MMMs, a majority of the ND was in the form of large-volume particles (10^4^–10^6^ nm^3^), while the small volume particles only occupy a minor fraction, which indicated the agglomeration of ND particles in the MMMs. On the other hand, the ND-PEI exhibited a more significant number of smaller particles (as measured by volume), indicating less agglomeration and better dispersion of ND-PEI in the polymer matrix due to the incorporation of PEI.

### 3.3. Gas Separation Performance

**Figure 6:**
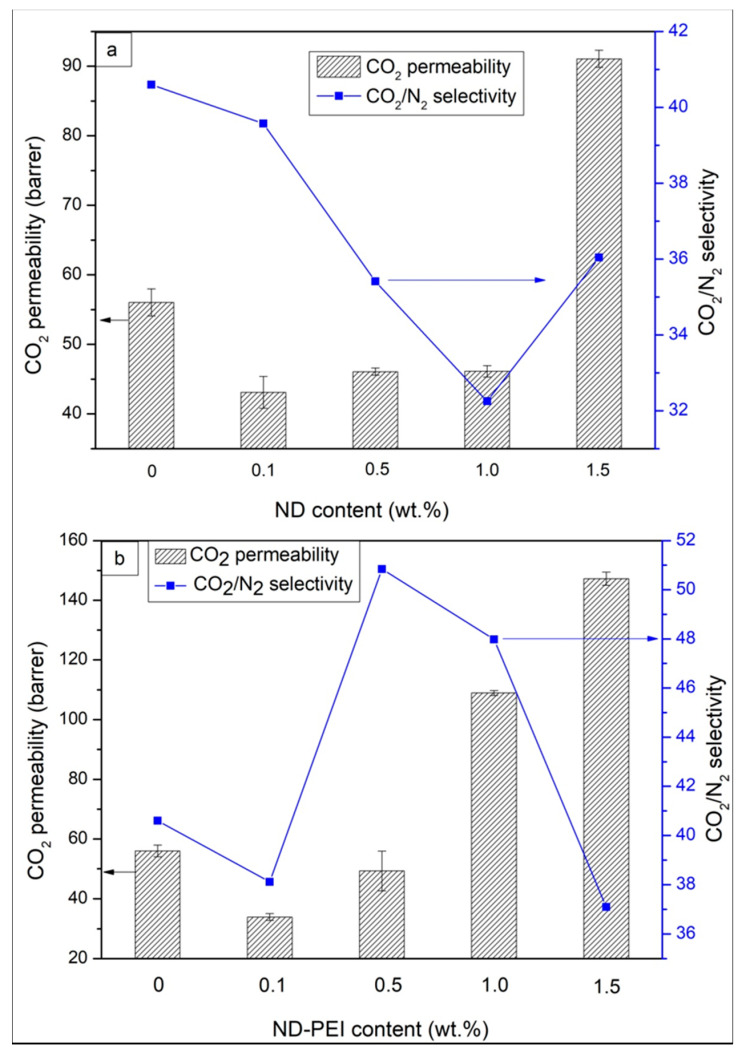
Gas separation performance of Pebax/ND MMMs (**a**) and Pebax/ND-PEI MMMs (**b**).

The ideal gas separation performance of the pristine Pebax membranes, Pebax/ND MMMs and Pebax/ND-PEI MMMs were investigated by single gas permeability measurements and the results are shown in Figure 6 and Table S1. The pristine Pebax membrane showed a CO_2_ permeability of 56 barrer with a CO_2_/N_2_ selectivity of 40.60, which is consistent with a previous study [45]. At a lower loading of ND (0.1 wt.%, 0.5 wt.%, 1 wt.%), the CO_2_ and N_2_ permeability of ND MMMs decreased after the introduction of ND when compared to the neat Pebax membranes, with the CO_2_/N_2_ selectivity reduced simultaneously. The presence of ND in the polymer matrix may occupy free volume between the polymer chains, which combined with the non-porous nature of ND, prevented gas diffusion through the membrane, leading to a reduction in gas permeability. Further, non-selective voids may form in the ND aggregates due to poor filler dispersion, resulting in the deterioration of CO_2_/N_2_ selectivity of the MMMs. At 1.5 wt.% loading of ND, the Pebax/ND MMMs exhibited a higher CO_2_ permeability as well as better CO_2_/N_2_ selectivity compared to the lower ND loading MMMs. At high loading, ND particles tend to form big clusters of agglomeration, which disrupted the polymer structure and created the non-selective voids inside the MMMs that in turn increase the permeability of both CO_2_ and N_2_ through the membranes.

In the case of MMMs with ND-PEI (Figure 6), at 0.1 wt.% loading of ND-PEI, the gas permeability of MMMs decreased while the CO_2_/N_2_ selectivity slightly reduced. In this case, the incorporation of PEI may improve the interfacial adhesion of ND and the Pebax matrix, which results in the reduction of free volume in the MMMs due to the occupation of ND-PEI particles. This result leads to the deterioration of both CO_2_ and N_2_ permeability through the MMMs. At lower loading of ND-PEI, the amount of PEI incorporated in the MMMs may not be enough to enhance the CO_2_ permeability to overcome the deterioration above, leading to a slight reduction in the case of MMMs with ND-PEI action in CO_2_/N_2_ selectivity. When the loading of ND-PEI is increased (up to 1 wt.%), both CO_2_ permeability and CO_2_/N_2_ selectivity of the Pebax/ND-PEI MMMs were improved compared to the pristine Pebax. The introduction of the PEI layer significantly enhanced the interfacial interaction between ND particles and polymer matrix, which prevents the formation of aggregates and reduces the formation of non-selective voids, leading to better CO_2_/N_2_ separation performance. Furthermore, the PEI layer on the ND surface can also act as the “CO_2_ carrier agent” due to the amine functional groups, which further enhances the solubility of CO_2_ through the MMMs and subsequently improves the CO_2_/N_2_ selectivity. At 1.5 wt.% loading of ND-PEI, the MMMs show higher permeability for both CO_2_ and N_2_ while the CO_2_/N_2_ decreased (37.09), which can be due to the slight formation of the ND aggregations caused by the higher concentration of ND in the polymer matrix. This is in agreement with the SEM investigation. We conclude that depositing PEI on ND presents a modification method to solve the nano-filler aggregation problem and improve the filler/polymer interface, leading to a significant improvement in membrane performance.

### 3.4. Modelling

**Figure 7:**
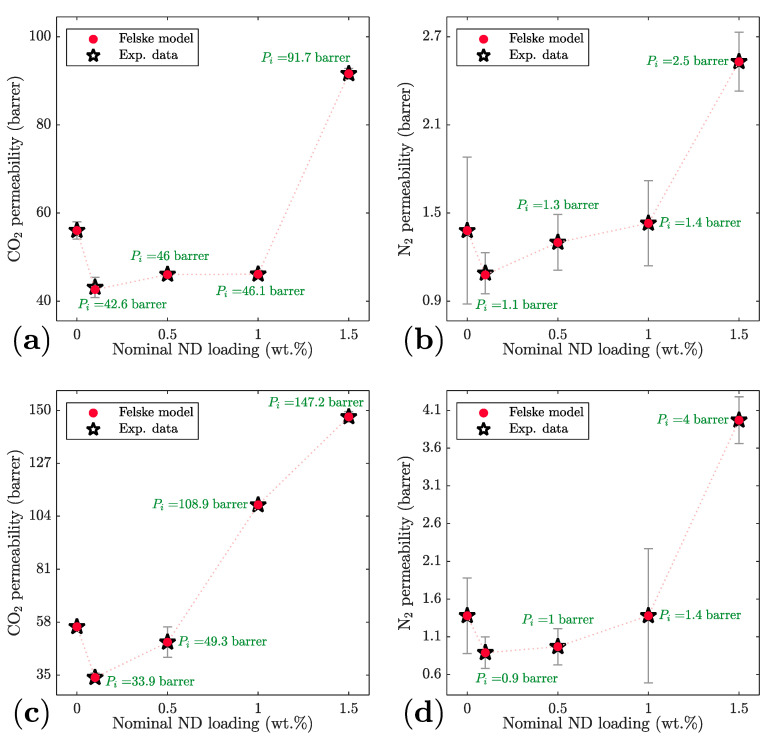
Predicted CO_2_ and N_2_ MMM and interfacial permeabilities with increase of ND loading. (**a**) CO_2_ in Pebax/ND, (**b)** N_2_ in Pebax/ND, (**c**) CO_2_ in Pebax/ND-PEI (**d**) N_2_ in Pebax/ND-PEI. Dotted lines correspond to a guide for the eye. Error bars correspond to experimental error bars.

Finally, because Figure 5 and Figure 6 suggest that surface modification of ND with PEI improves the filler/polymer interfacial properties, leading to a significant improvement in the MMM performance, the interfacial permeabilities (*P_i_*) of each gas are calculated using the Felske model [31] summarized in Section 2.6. This model has been shown to provide a good approximation of interfacial properties (i.e., interface thickness and permeability) when particle loading is low (less than 10–15%) in the MMM, as discussed elsewhere [32,33]. Figure 7 depicts a comparison of the experimental permeabilities (open star symbols) to those based on the Felske model (closed circle symbols) with an increase of ND loading in the MMM. Figure 7a,b depicts CO_2_ and N_2_ permeabilities in the Pebax/ND MMMs while Figure 7c,d depicts those in Pebax/ND-PEI MMMs, respectively. It is noted that while abscissas in Figure 7 correspond to the nominal ND percentage weight fraction (c.f. Section 2.3), pristine Pebax and ND particle densities are used to calculate the ND volume fraction in the MMMs and thus solve the Felske model for the interfacial permeability $\left(P_{i}\right)$ in each case (c.f. Section 2.6). Further, each theoretical data point is accompanied by the calculated *P_i_* considering the ND permeability equal $P_{f}=0$ (non-porous filler) and a ratio of the interface thickness to ND size (radius) of $l_{i}/r_{o}=40$ in the Felske model. For this membrane system, the interfacial permeabilities are found to be insensitive to the variation of this ratio, as shown in the Supplementary Material (Figure S5) for $l_{i}/r_{o}$ between 20–1000, with no suitable model solution found for ratios below 20. This tendency suggests that the ratio of the interface thickness to the particle ratio is likely to be greater than 20 for this system. While this result is in agreement with recently reported ND structures [46,47], having ND core sizes relatively smaller than their shells, detailed characterisation of the Pebax/ND-PEI interfacial morphology will be required to estimate the interface thickness quantitively. In Figure 7a,b the interfacial permeabilities are slightly lower than those in neat Pebax at low loadings (0.1 wt.%, 0.5 wt.%, and 1.0 wt.%). A similar tendency is depicted in Figure 7c,d at low loadings (0.1 wt.% and 0.5 wt.%) for both gases in Pebax/ND-PEI MMMs, which suggests the absence of interfacial voids for these membranes. Alternatively, the CO_2_ and N_2_ interfacial permeabilities in Figure 7a,b for the highest ND loading (1.5 wt.%) are about twice those in the neat Pebax membranes, which corroborates the presence of non-selective interfacial voids in the MMM due to particle agglomeration. This tendency has also been recently recognised in the removal of lead ions from aqueous solutions in nanodiamond/chitosan-polyvinyl alcohol (ND/CS-PVA) membranes, in which particle agglomeration was observed for ND loadings of 1.5 wt.% [48]. Further, while the CO_2_ permeability in Figure 7c is also about twice that in the neat polymer when the ND loading is 1.0%, N_2_ in Figure 7d is about the same as in the neat Pebax membrane. This tendency corroborates that the increase of the CO_2_/N_2_ selectivity, when the ND surface is modified with PEI, is associated with an increase in the solubility of CO_2_ due to the presence of PEI amine functional groups, which improve the affinity to CO_2_ without enhancing the N_2_ adsorption capacity. Alternatively, when ND loading is 1.5 wt.% in Figure 7c,d both CO_2_ and N_2_ permeabilities are significantly larger than in neat Pebax, which is associated with a combination of effects in the membrane system. On one hand, the CO_2_ has a better affinity for the PEI amine functional groups, which leads to an increase of the interfacial permeability. On the other, this MMM exhibited particle agglomeration, leading to the formation of non-selective interfacial voids and thus a further increase of both CO_2_ and N_2_ interfacial permeabilities. 

## 4. Conclusions

In this study, mixed matrix membranes based on ND nanoparticles and Pebax matrix for CO_2_/N_2_ separation were obtained. Surface modification of ND by PEI decoration has been conducted, of which success was confirmed by elemental analysis, XPS and FTIR. The presence of PEI layer on ND surface effectively improved the interfacial adhesion and dispersion of ND in the Pebax matrix, which were clearly indicated by SEM observation. The ideal selectivity of CO_2_/N_2_ was also significantly improved with the incorporation of PEI up to 1 wt.% of ND-PEI filler in the MMMs, due to the “CO_2_ carrier” role of PEI, which was corroborated through the calculation of the CO_2_ and N_2_ interfacial permeabilities using the Felske model. These results expressed an effective modification approach for applying the potential non-porous ND into gas separation membrane application. Moreover, the PEI decoration method can be comparable with a wide range of nanofillers for improved distribution in a polymer matrix.

## Figures and Tables

**Table 1:** Elemental weight ratio of pristine ND and ND-PEI particles.

Samples	Elemental Ratio (wt.%)		
	N	C	H
ND	1.61	86.27	0.64
ND-PEI	4.34	83.94	1.25

## Supplementary Materials

The following are available online at https://www.mdpi.com/article/10.3390/membranes11050328/s1, Figure S1. Typical FIB–SEM images of Pebax/ND 1.5 wt.% MMM: (a) FIB milling trend and (b) cross-sectional image in BSE mode, Figure S2. Thermogravimetric analysis of ND–PEI at a ramp rate of 20 °C min-1, Figure S3. Fourier-transform infrared spectra of ND, ND–PEI and PEI., Figure S4. Membrane cross–section SEM images of pristine Pebax (a, b), Pebax/ND 1.5 wt.% (c, d) and Pebax/ND–PEI 1.5 wt.% (e, f) MMMs. Figure S5. Predicted CO2 and N2 interfacial and MMM permeabilities with increase the ratio of the interface thickness to particle size. (a) CO2 interfacial permeability (b) CO2 permeability in Pebax/ND-PEI and, (c) N2 interfacial permeability (d) N2 permeability in Pebax/ND-PEI., Table S1. Gas permeability and selectivity of pure Pebax membrane, Pebax/ND MMMs and Pebax/ND–PEI MMMs.
