# Supplementary material for: Mitigating the Agglomeration of Nanofiller in a Mixed Matrix Membrane by Incorporating an Interface Agent
Source: Membranes (Basel). 2021 Apr 29;11(5):328. doi: 10.3390/membranes11050328 (PMC8145898; doi:10.3390/membranes11050328)
Supplement: Supplementary file 1 [file membranes-11-00328-s001.zip › supplementary membranes-1176938.pdf]

membranes

Supplementary Materials

Mitigating the Agglomeration of Nanofiller in a Mixed Matrix Membrane by Incorporating an Interface Agent

Manh-Tuan Vu [1,2], Gloria M. Monsalve-Bravo [1], Rijia Lin [1,*], Mengran Li [1], Suresh K. Bhatia [1], Simon Smart [1,3,*]

[1] School of Chemical Engineering, The University of Queensland, Brisbane, QLD 4072, Australia; manh.vu@uq.net.au (M.-T.V.); g.monsalvebravo@uq.edu.au (G.M.M.-B.); m.li6@uq.edu.au (M.L.); s.bhatia@uq.edu.au (S.K.B.)

[2] Institute for Tropical Technology, Vietnam Academy of Science and Technology, Hanoi 100000, Vietnam

[3] Dow Centre for Sustainable Engineering Innovation, The University of Queensland, Brisbane, QLD 4072, Australia

* Correspondence: r.lin1@uq.edu.au (R.L.); s.smart@uq.edu.au (S.S.)

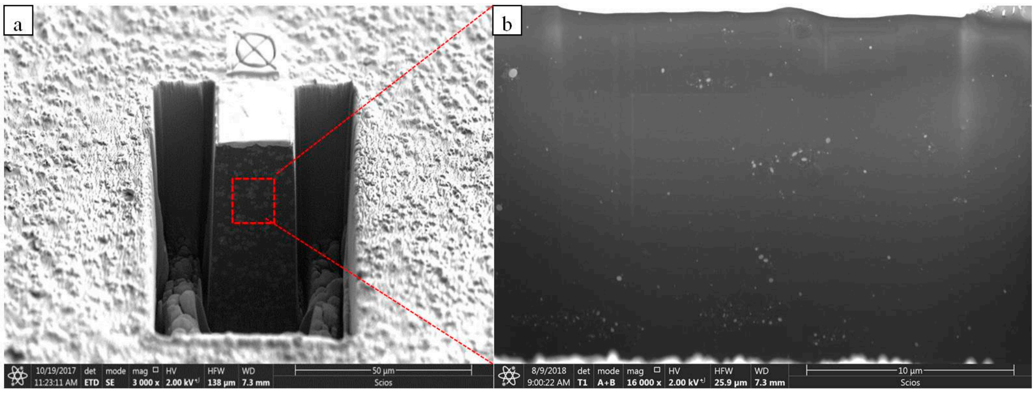

Figure S1. Typical FIB–SEM images of Pebax/ND 1.5 wt.% MMM: (**a**) FIB milling trend and (**b**) cross-sectional image in BSE mode.

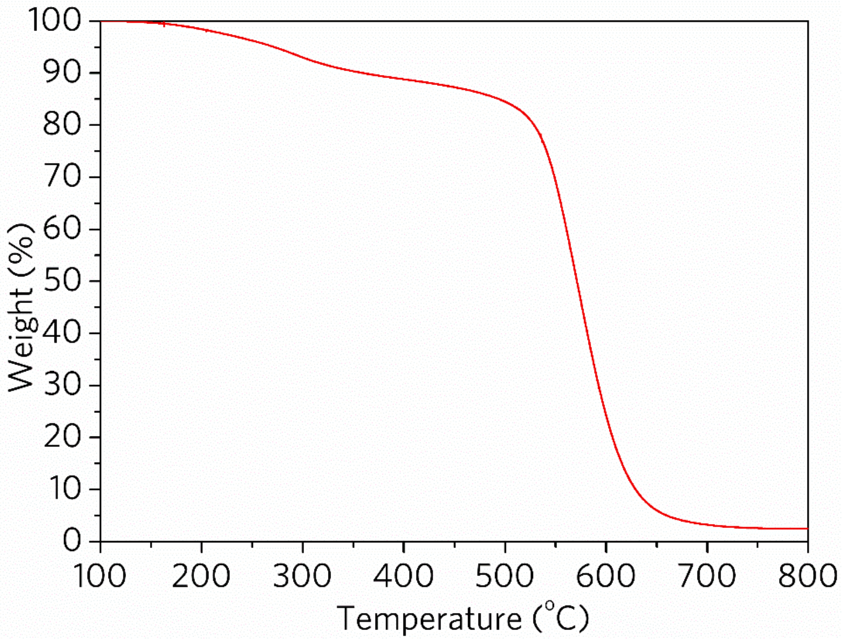

Figure S2. Thermogravimetric analysis of ND–PEI at a ramp rate of 20 °C min^{-1}.

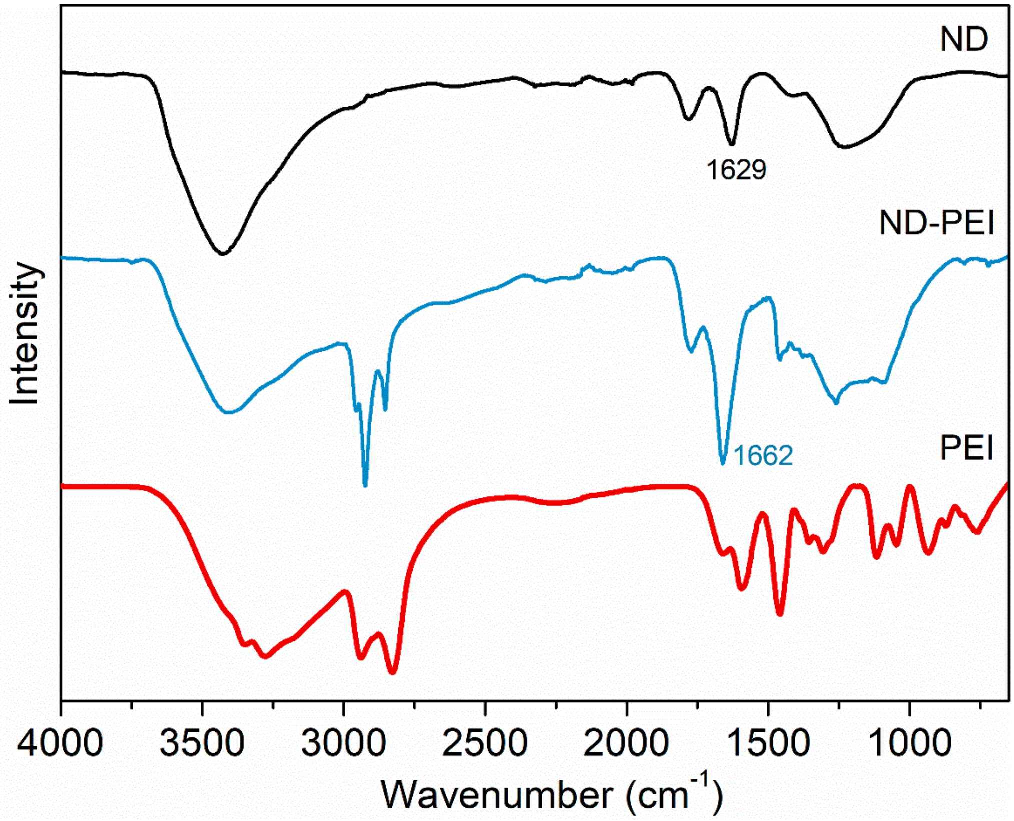

Figure S3. Fourier-transform infrared spectra of ND, ND–PEI and PEI.

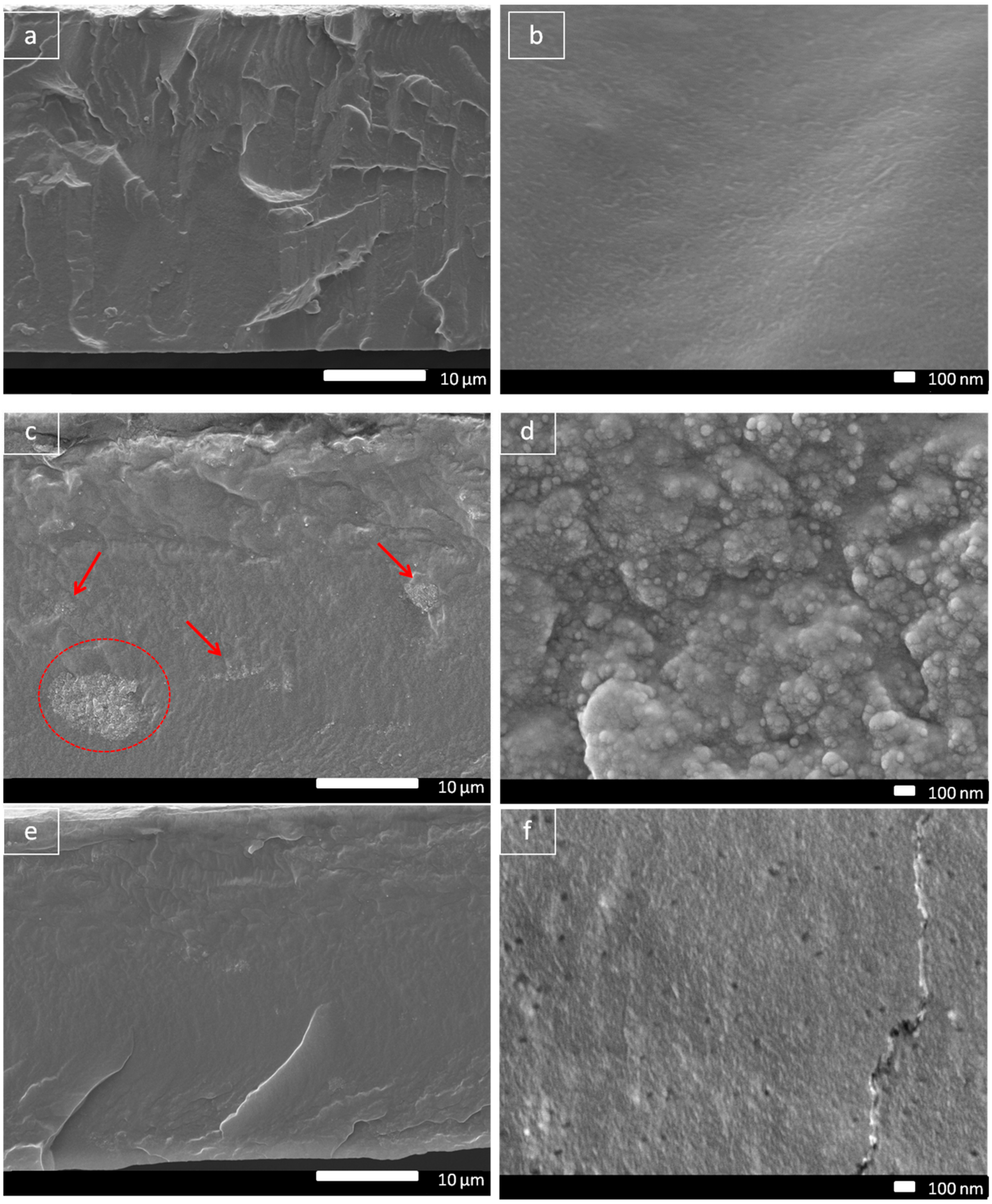

Figure S4. Membrane cross–section SEM images of pristine Pebax (**a, b**), Pebax/ND 1.5 wt.% (**c, d**) and Pebax/ND–PEI 1.5 wt.% (**e, f**) MMMs.

Predicted CO_2 and N_2 interfacial permeabilities with increase of the ratio of the interface thickness to particle size

Figure S5 depicts predicted CO_2 and N_2 interfacial and MMM permeabilities with increase in the ratio of the interface thickness to particle size (ℓ_i/r_o) for a Pebax/ND-PEI MMM with a nominal ND loading of 1.0 wt.%. In Figure S5a, c the mean CO_2 and N_2 interfacial permeabilities are shown in the right corner. In Figure S5b, d the continuous line correspond to the CO_2 and N_2 experimental permeabilities while the dotted lines correspond to one standard deviation of the experimental data point. In both cases, the closed circle symbol corresponds to ℓ_i/r_o in which the Felske model prediction deviates beyond the experimental error.

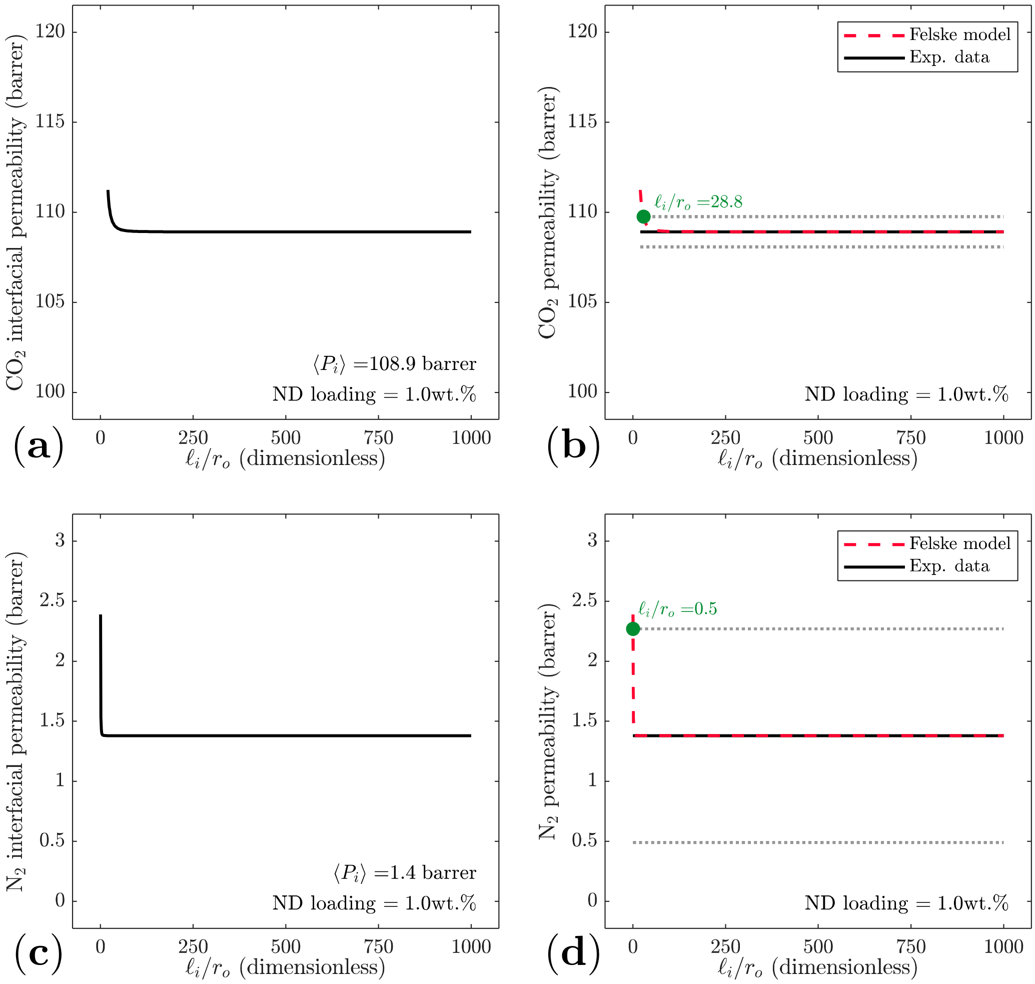

Figure S5. Predicted CO_2 and N_2 interfacial and MMM permeabilities with increase the ratio of the interface thickness to particle size. (**a**) CO_2 interfacial permeability (**b**) CO_2 permeability in Pebax/ND-PEI and, (**c**) N_2 interfacial permeability (**d**) N_2 permeability in Pebax/ND-PEI.

Table S1. Gas permeability and selectivity of pure Pebax membrane, Pebax/ND MMMs and Pebax/ND–PEI MMMs.

**Sample**	**Permeability (barrer)**		**Selectivity**
	CO_2	N_2	CO_2/N_2
Pebax	56.03±1.96	1.38±0.50	40.60
Pebax/ND 0.1 wt.%	43.12±2.29	1.09±0.14	39.57
Pebax/ND 0.5 wt.%	46.08±0.53	1.30±0.19	35.41
Pebax/ND 1.0 wt.%	46.12±0.84	1.43±0.29	32.25

Pebax/ND 1.5 wt.%	91.06±1.23	2.53±0.21	36.04
Pebax/ND-PEI 0.1 wt.%	33.92±1.11	0.89±0.21	38.11
Pebax/ND-PEI 0.5 wt.%	49.31±6.63	0.97±0.24	50.84
Pebax/ND-PEI 1.0 wt.%	108.92±0.84	2.27±0.89	47.98
Pebax/ND-PEI 1.5 wt.%	147.23±2.21	3.97±0.31	37.09
